# Computed Tomography Imaging of Thoracic Aortic Surgery: Distinguishing Life-Saving Repairs from Life-Threatening Complications

**DOI:** 10.3390/jimaging11040119

**Published:** 2025-04-17

**Authors:** Marco Fogante, Paolo Esposto Pirani, Fatjon Cela, Jacopo Alfonsi, Corrado Tagliati, Liliana Balardi, Giulio Argalia, Marco Di Eusanio, Nicolò Schicchi

**Affiliations:** 1 Maternal-Child, Senological, Cardiological Radiology and Outpatient Ultrasound, Department of Radiological Sciences, University Hospital of Marche, 60126 Ancona, Italy; paolo.espostopirani@ospedaliriuniti.marche.it (P.E.P.); fatjon.cela@ospedaliriutini.marche.it (F.C.); argalia.giulio@ospedaliriuniti.marche.it (G.A.); nicolo.schicchi@ospedaliriuniti.marche.it (N.S.); 2 Cardiac Surgery Unit, Department of Cardiovascular Sciences, University Hospital of Marche, 60126 Ancona, Italy; jacopo.alfonsi@ospedaliriuniti.marche.it (J.A.); marco.dieusanio@ospedaliriuniti.marche.it (M.D.E.); 3 Ospedale di Comunità Maria Montessori di Chiaravalle, AST Ancona, 60033 Chiaravalle, Italy; corrado.tagliati@gmail.com; 4 Health Professions Area, Diagnostic Technical Area, University Hospital of Marche, 60126 Ancona, Italy; liliana.balardi@ospedaliriuniti.marche.it; 5 Cardiovascular Radiological Diagnostics, Department of Radiological Sciences, University Hospital of Marche, 60126 Ancona, Italy

**Keywords:** aorta, computed tomography, CTA, aortic pathology, aortic surgery, postoperative monitoring

## Abstract

Thoracic aortic pathology encompasses a spectrum of life-threatening conditions that demand prompt diagnosis and intervention. Significant advancements in surgical management, including open repair, endovascular aortic repair, and hybrid techniques, have markedly enhanced patient outcomes. However, these procedures necessitate meticulous imaging follow-up to identify potential complications. Computed tomography angiography remains the gold standard for evaluating aortic pathology, guiding surgical planning, and monitoring postoperative changes. A thorough understanding of the characteristic imaging features associated with various aortic surgical techniques is crucial for precise assessment, enhancing postoperative surveillance, and optimizing patient management. Distinguishing between surgical complications and postoperative findings is vital to prevent misdiagnosis. This review examines the imaging characteristics of thoracic aortic diseases and their corresponding surgical interventions, emphasizing the differentiation between expected postoperative findings and true pathological conditions. This approach aims to facilitate accurate diagnosis and effective management of complications, ultimately improving patient care.

## 1. Introduction

Thoracic aortic pathology encompasses a wide range of potentially fatal conditions, each varying in presentation depending on the affected anatomical segment [1]. Prompt diagnosis and appropriate management are critical, as delayed intervention can lead to catastrophic complications such as rupture, ischemia, or organ malperfusion [2].

Over the years, advancements in both open surgical repair and endovascular techniques have significantly improved patient outcomes, allowing for tailored treatment approaches based on the extent and severity of the disease. However, these interventions require meticulous preoperative planning and rigorous postoperative imaging surveillance to detect and manage complications [3].

Computed tomography angiography (CTA) is the gold standard for evaluating thoracic aortic disease, providing exceptional anatomical detail and high spatial resolution. It is essential for assessing disease extent, guiding surgical planning, and identifying postoperative complications that may require urgent intervention. As endovascular procedures become increasingly common and lifelong imaging follow-up is required, radiologists must master the interpretation of CTA findings to accurately distinguish normal postoperative changes from true pathological conditions [4,5].

This review provides a comprehensive analysis of thoracic aortic diseases, surgical interventions, and their complications, with an emphasis on differentiating true pathology from expected postoperative changes. By highlighting key imaging features on CTA, this approach aims to enhance the radiologist’s ability to diagnose, monitor, and manage these complex cases effectively.

## 2. Methodology

This review was conducted using a targeted literature search of PubMed, Embase, and Google Scholar for studies published from January 2000 to January 2024. The search used combinations of keywords such as “thoracic aortic surgery”, “postoperative CTA”, “graft complications”, “aortic imaging pitfalls”, “Bentall”, “Cabrol”, and “TEVAR complications”. We included peer-reviewed articles, clinical case series (including a minimum of 5 patients), systematic reviews, and major radiological society guidelines. Only English-language publications were included. Selection was driven by relevance to the differentiation between expected postoperative findings and pathological complications.

## 3. CT Imaging Protocol

Modern CT scanners generate high-resolution three-dimensional images of the thoracic aorta, with electrocardiogram (ECG) synchronization playing a crucial role in minimizing motion artifacts, particularly for precise assessment of the aortic root and its related structures [6].

The choice of ECG gating impacts both image quality and radiation exposure. Prospective ECG sequential gating captures images during a predefined phase of the cardiac cycle, optimizing radiation dose, while retrospective ECG gating continuously acquires helical CT data, allowing for phase selection but increasing radiation exposure. ECG-based tube current modulation helps to minimize dose while maintaining diagnostic quality. In specific cases, prospective high-speed, high-pitch helical scans with ECG triggering provide motion-free images of the aortic root during diastole but limit dynamic assessment [7].

For preoperative CTA or initial evaluation of an aortic root aneurysm, imaging must cover both systolic and diastolic phases to assess valve function and root geometry. This is achieved using retrospective ECG gating. Postoperative imaging for surveillance and for detecting complications can be performed with low-radiation techniques, such as prospective ECG sequential gating or prospective high-pitch helical scanning, focusing on reducing motion artifacts and ensuring reproducibility [6,8,9]. CTA datasets should be reviewed on dedicated workstations using multiplanar reformatting, maximum-intensity projections, curved planar reformation, and volume rendering to ensure accurate anatomical evaluation [10,11].

## 4. Structural and Zonal Anatomy of the Thoracic Aorta: From Histology to Surgical Landmarks

The aorta’s elasticity is determined by its three layers: the intima, media, and adventitia. The media, the thickest layer, contains lamellar units made up of elastic laminae, smooth muscle cells, collagen, and proteoglycans. With age, the ascending aorta and arch add new lamellae, while the descending aorta thickens existing ones. Muscle cells in the ascending aorta and arch come from neural crest cells, while those in the descending aorta arise from paraxial mesoderm, influencing their response to growth factors. The adventitia contains connective tissue, vasa vasorum, and lymphatic vessels, with vasa vasorum extending into the outer media [12].

The aortic root connects the left ventricle to the systemic circulation and is closely related to the pulmonary, mitral, and tricuspid valves. It includes the aortic annulus, a fibrous ring that supports the valve and serves as a graft attachment site. The aortic valve, typically tricuspid, can have anatomical variations like bicuspid or quadricuspid morphology. The commissures, where the leaflets attach to the aortic wall, are essential for valve function but may fuse in certain conditions. Below them, the interleaflet triangles extend from the left ventricular outflow tract. The sinuses of Valsalva are bulges in the root, with the right and left sinuses giving rise to the coronary arteries. The sinotubular junction marks the transition to the ascending aorta, ensuring proper valve function and circulatory stability [4]. The ascending aorta originates from the left ventricle, extends about 5 cm, and transitions into the aortic arch at the sternal angle (T4–T5). The aortic arch curves posteriorly and gives rise to three branches: the brachiocephalic artery (dividing into the right subclavian and right common carotid), the left common carotid artery, and the left subclavian artery. It transitions into the descending thoracic aorta at the aortic isthmus, a vulnerable area due to its fixation. The descending thoracic aorta supplies the thoracic organs before passing through the diaphragm to become the abdominal aorta [13].

The Ishimaru classification system divides the thoracic aorta into five zones (0 to 4), providing a structured approach to surgical and endovascular treatment based on anatomical landmarks and ideal stent graft landing zones (Table 1). Zone 0 includes the ascending aorta and the origin of the brachiocephalic artery. Due to the complex anatomy and the dynamic forces in this region, open surgical repair remains the preferred treatment, as endovascular options are technically challenging. Zone 1 includes the tract of the aortic arch with the origin of the left common carotid artery. When stent placement is considered in this region, an extraanatomical revascularization may require using the right common carotid artery or right subclavian artery as the donor vessels. Zone 2 includes the tract of the aortic arch with the origin of the left subclavian artery. Because of the involvement of critical arch vessels, hybrid repair techniques are commonly employed, often incorporating an extraanatomical revascularization with left carotid-subclavian bypass or transposition. Zone 3 includes the proximal descending thoracic aorta. This region serves as a primary landing zone for thoracic endovascular aortic repair (TEVAR), which is frequently used for treating aneurysms and dissections. Zone 4 encompasses the distal descending thoracic aorta, continuing toward the diaphragm. TEVAR is the most common intervention in this area, but open surgical repair may be necessary for complex or extensive disease involving multiple segments. By defining these zones, the Ishimaru classification helps guide the selection of the most appropriate treatment approach, whether open surgery, hybrid procedures, or purely endovascular techniques [14].

## 5. Thoracic Aortic Surgical Pathology: CT Imaging Features and Diagnostic Insights

An aortic aneurysm is an abnormal dilation of the aorta that significantly increases the risk of rupture. Aortic root aneurysms, often linked to hypertension, atherosclerosis, or genetic disorders such as Marfan or Ehlers–Danlos syndrome, are characterized on CT by an aortic diameter exceeding 4 cm and may be associated with aortic regurgitation due to valve distortion. Thoracic aortic aneurysms, whether focal or diffuse, appear on CTA as dilations greater than 50% of the normal vessel diameter, often accompanied by mural thrombus or calcifications, which can further complicate disease progression and management [15,16,17].

Aortic pseudoaneurysms are contained ruptures of the aortic wall, which often occur due to trauma, infection, surgery, or chronic dissection. On CTA, they appear as irregular, sac-like outpouchings with a narrow neck, often with wall thickening, periaortic hematoma, or contrast extravasation. When at the aortic root, they may compress nearby structures, risking coronary involvement or aortic regurgitation, requiring urgent evaluation [18].

Acute aortic syndrome is a life-threatening condition involving a tear or rupture in the aortic wall, including aortic dissection, penetrating atherosclerotic ulcer (PAU) and intramural hematoma (IHM). Aortic dissection occurs when blood enters between the layers of the aortic wall, forming a true and false lumen, which may be dilated or contain thrombus. Type A dissection, affecting the ascending aorta, requires urgent surgery due to the risk of coronary involvement, myocardial infarction, or cardiac tamponade. Type B dissection, involving the descending aorta, is typically managed medically unless complications like ischemia develop [18]. PAU and IMH are two distinct but related acute aortic syndromes that can lead to severe complications if not promptly diagnosed and managed. A PAU occurs when an atherosclerotic plaque erodes through the internal elastic lamina, creating an ulcer that extends into the aortic media. On CTA, it appears as contrast-filled outpouchings or crater-like defects in the aortic wall, often associated with surrounding thickening or enhancement, indicating inflammation. In contrast, an IMH results from bleeding within the aortic media, typically due to the rupture of the vasa vasorum without an intimal tear. CTA findings of IMH include a crescent-shaped or circular hyperattenuating area along the aortic wall without contrast enhancement, distinguishing it from a true aortic dissection [19,20].

Thoracic aortic rupture is a life-threatening condition often resulting from trauma, hypertension, or aortic aneurysms. It involves the disruption of the aortic wall, leading to massive internal bleeding. On CT imaging, findings typically include a large hematoma or retroperitoneal hemorrhage, with evidence of an intimal flap or aortic dissection. The aorta may appear dilated, and there may be contrast extravasation suggesting active bleeding. The presence of aortic wall irregularities or contour changes can further indicate rupture, and a CTA can help assess the extent of the injury and surrounding organ involvement [21].

**Figure 1:**
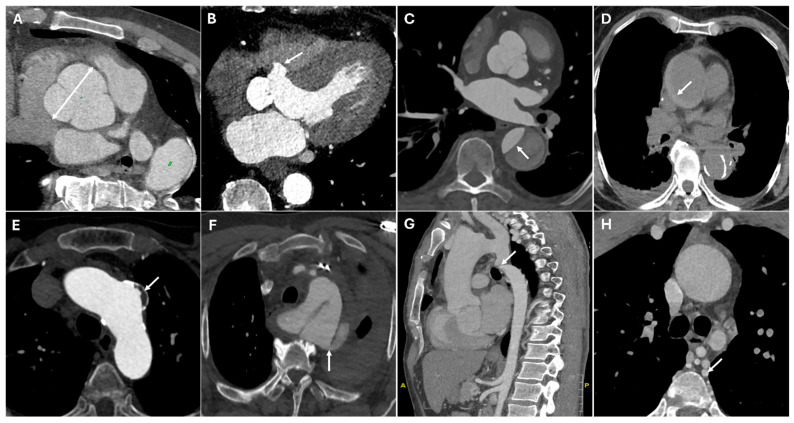
CT images of thoracic aortic surgical pathologies. Panel (**A**) shows a large aortic root aneurysm (double arrow). Panel (**B**) depicts an aortic root pseudoaneurysm caused by valve infection (white arrow), a contained rupture resulting from a defect in the aortic wall. Panel (**C**) illustrates an intimal flap in an acute aortic type B dissection (white arrow). Panel (**D**) demonstrates an intramural hematoma (white arrow), a hyperdense collection of blood within the aortic wall. Panel (**E**) highlights a pseudoaneurysm of the aortic arch caused by penetrating atherosclerotic ulcer (white arrow), where ulceration of an atherosclerotic plaque extends into the media. Panel (**F**) reveals a thoracic aortic traumatic rupture with massive hemorrhage (white arrow). Panels (**G**,**H**) display a coarctation of the aorta (white arrow), a congenital narrowing of the descending thoracic aorta, along with collateral circulation (white arrow), which develops to compensate for restricted blood flow.

Coarctation of the aorta is a congenital narrowing, usually diagnosed in childhood, leading to hypertension and collateral vessel formation. CT imaging reveals a focal aortic narrowing with post-stenotic dilation and collateral circulation, especially in the intercostal arteries [22]. Figure 1 presents CT images illustrating various surgical pathologies of the thoracic aorta.

## 6. Surgical Management of the Thoracic Aorta: Techniques, CT Imaging, and Postoperative Assessment

**Figure 2:**
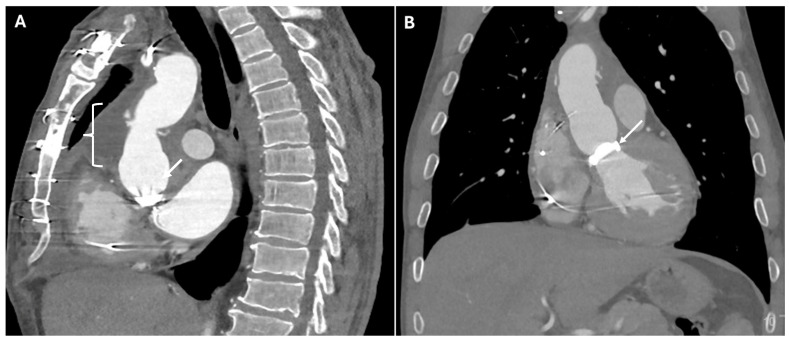
CT images of Bentall procedure. These images illustrate the typical postoperative appearance of the Bentall procedure. Panel (**A**) depicts a tubular graft replacing the aortic root and ascending aorta (white opening brace) with the prosthetic aortic valve (white arrow). The graft appears as a well-defined, high-attenuation structure with smooth walls, seamlessly integrated into the native aortic anatomy. Panel (**B**) highlights the prosthetic aortic valve (white arrow) within the composite graft. The valve’s distinct radiodense structure is visible, positioned at the level of the aortic annulus.

Ascending aorta pathologies are usually treated with open surgical repair. Surgical approaches to open aortic repair include the inclusion and interposition techniques. The inclusion technique involves suturing the native aorta around the graft, creating a perigraft space. Advancements in surgical methods have led to the predominance of the interposition technique, in which the diseased aorta is completely excised and replaced with a graft [23]. The Bentall procedure is the traditional method for replacing the aortic root and ascending aorta, utilizing a composite graft that contains either a mechanical or bioprosthetic valve. The coronary arteries are reimplanted into the graft, with a modified technique often employing a coronary button to improve the anastomosis. This procedure is commonly performed for conditions like aortic root aneurysms, annuloaortic ectasia, and Type A aortic dissections. On CT imaging, the tubular graft replacing the aortic root and ascending aorta is clearly visualized, along with the coronary reimplantation sites and the prosthetic valve (Figure 2) [24].

**Figure 3:**
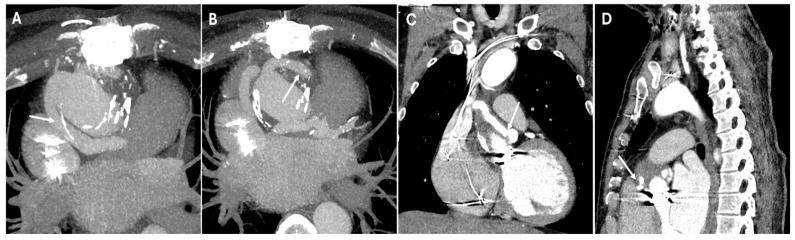
CT images of Cabrol procedure. Panels (**A**,**B**) show the parallel interposed conduit, known as the Cabrol graft (white arrows), which is used to reimplant the coronary arteries into the new aortic graft. Panels (**C**,**D**) illustrate the anastomotic sites (white arrows) between the Cabrol graft and the left and right coronary arteries, demonstrating the surgical connections that restore coronary perfusion.

In situations where direct coronary reimplantation is not possible, the Cabrol procedure offers an alternative. This technique uses a prosthetic conduit to connect the coronary ostia to the aortic graft, providing a viable solution for patients with severe atherosclerosis or those requiring complex reoperations. On CT, the image typically shows a graft replacing the aortic root and ascending aorta. In addition to the aortic graft, a parallel interposed conduit—referred to as the Cabrol graft—connects the coronary arteries to the new aortic graft (Figure 3).

The Yacoub procedure involves resecting the diseased aortic root and reconstructing the sinuses of Valsalva with a scalloped synthetic graft. This approach preserves native valve function, though it lacks annular support, which may lead to annular dilation and progressive valve insufficiency over time. The David procedure replaces the aortic root while reimplanting the native valve into a supportive graft, preventing annular dilation and ensuring long-term valve stability. The David procedure offers superior long-term durability compared to the Yacoub procedure due to its reinforcement of the aortic annulus, reducing the risk of late aortic regurgitation. This procedure is especially beneficial for younger patients, particularly those with connective tissue disorders like Marfan syndrome. In contrast, the Yacoub procedure preserves the flexibility of the aortic root, which may be advantageous in select cases [25].

Aortic arch replacement is indicated when pathology extends beyond the ascending aorta, with several surgical techniques available for repair. For less extensive interventions, hemiarch repair extends from zone 0 reconstruction to the lesser curvature of the aortic arch. The grafts used in this approach feature a tongue-shaped distal portion, which is visible on multiplanar CT reconstructions, and are often reinforced with sutures or felt for added stability. For complex aortic arch disease, hybrid procedures are commonly employed, combining open surgery with endovascular interventions. These repairs are categorized based on anatomical complexity. Type I hybrid repair involves debranching of the arch vessels, with a stent landing in zone 0. Type II hybrid repair integrates open arch surgery, characterized by a surgical graft in zone 0, with a stent landing in the same zone. Type III hybrid repair—potentially an elephant trunk type—incorporates open surgical grafting extending from zone 0 to zones 1, 2, or 3, with a stent landing within the open graft. On CT, surgical grafts appear as well-defined, tubular structures with uniform contrast enhancement and smooth walls, whereas endovascular stent grafts are seen as high-density metallic structures conforming to the aortic lumen.

Understanding arch repair also requires knowledge of how arch vessels are managed. One approach, the island patch technique, involves excising the arch vessels along with a portion of surrounding native aortic tissue and reattaching them “en bloc” to an opening in the surgical graft. On CT, this repair is identified by the characteristic piggyback appearance of the island, particularly in cases of continued aortic dilatation. Another approach, the debranching technique, relocates the arch vessels using separate tubular grafts connected to the ascending aorta or an aortic graft. This method often incorporates additional bypasses, such as left carotid-to-left subclavian or carotid-to-carotid connections. On CT, these repairs are recognized by the non-anatomic orientation of the branched grafts, with more proximal attachments on the aortic graft. Distinct caliber changes at the anastomotic sites and the presence of reinforcement materials further characterize these reconstructions. A third strategy utilizes a prefabricated branched graft with individual extensions for each arch vessel, facilitating complete aortic arch replacement. On CT, these grafts are identified by their anastomoses with the remaining portions of the arch vessels, frequently accompanied by caliber changes and reinforcement materials. Endovascular advances allow for complete aortic arch repairs using chimney or snorkel stenting, where parallel stent grafts extend from the arch vessels alongside an endovascular graft. Some stent grafts feature fenestrations or branches to maintain blood flow to the arch vessels. On CT, these techniques appear as extended stent grafts with preserved communication to the arch vessels [3,5,23,25].

**Figure 4:**
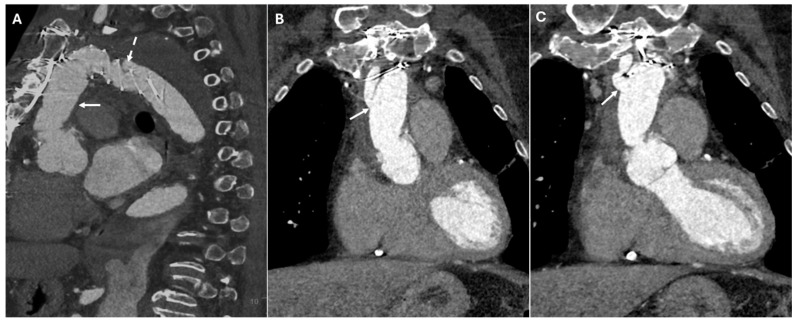
CT images of the frozen elephant trunk. These images provide a comprehensive view of the frozen elephant trunk procedure, which combines open surgical and endovascular techniques to treat extensive thoracic aortic disease, particularly in cases involving the aortic arch and descending aorta. Panel (**A**) shows the open proximal graft (white arrow) and the distally extended stent (dashed line). Panels (**B**,**C**) depict the arch vessels connected to the aortic graft (white arrows). These demonstrate the anatomical relationships and patency of the revascularized supra-aortic branches, which are crucial for maintaining adequate cerebral and upper-extremity perfusion.

Many repairs extend beyond the aortic arch into zones 3 and 4, presenting unique surgical challenges. In cases involving the descending thoracic aorta, the elephant trunk technique enables a staged repair of extensive aortic disease. This procedure traditionally consists of two stages. The first stage involves open surgical repair of the ascending aorta and arch, with a graft extending distally into the descending aorta. On a CT scan, this appears as a floating tubular graft within the descending aortic lumen, often showing two parallel hypoattenuating linear structures, sometimes with metallic clips at the distal tip. It is essential to differentiate this from an intimal flap, which lacks the parallel configuration and reinforcement materials. In the second stage, the repair is completed through an open thoracotomy, replacing the descending aorta and connecting a new graft to the previously implanted elephant trunk. Modern hybrid techniques have largely replaced this staged approach by incorporating an endovascular stent within the pre-existing elephant trunk graft, which is known as the frozen elephant trunk technique. On a CT scan, this appears as a seamless transition between the open proximal graft and the distally extended stent, providing durable fixation within the descending aorta (Figure 4).

Conversely, the reverse elephant trunk technique reverses the sequence, beginning with endovascular stenting of the descending aorta, followed by open arch replacement in a second stage. A variation, the buffalo trunk technique, integrates an open arch graft with an endovascular stent, allowing for a single-stage repair of both the aortic arch and descending aorta [26,27,28].

Thoracic descending aortic pathologies can be managed with open surgical repair, TEVAR, or hybrid techniques. Open repair involves resection of the diseased segment and replacement with a surgical graft, which appears on CT as a well-defined, tubular structure with uniform contrast enhancement and smooth walls. TEVAR deploys a stent graft to exclude the aneurysm or dissection, which appears as a high-density metallic structure conforming to the aortic lumen, ensuring complete exclusion of pathology. Hybrid approaches combine both techniques, appearing on CT as a mixture of bypass grafts and endovascular components [29]. Table 2 summarizes the surgical management of the thoracic aorta [7].

## 7. Postoperative Complications in Thoracic Aortic Surgery: CT Imaging and Diagnosis

Postoperative CTA plays a crucial role in assessing graft and valve integrity, and in the early detection of these complications. Postoperative complications following prosthetic and hybrid procedures include coronary artery complications, anastomotic dehiscence, leaks, pseudoaneurysm, infection of perigraft fluid collection, and tamponade.

**Figure 5:**
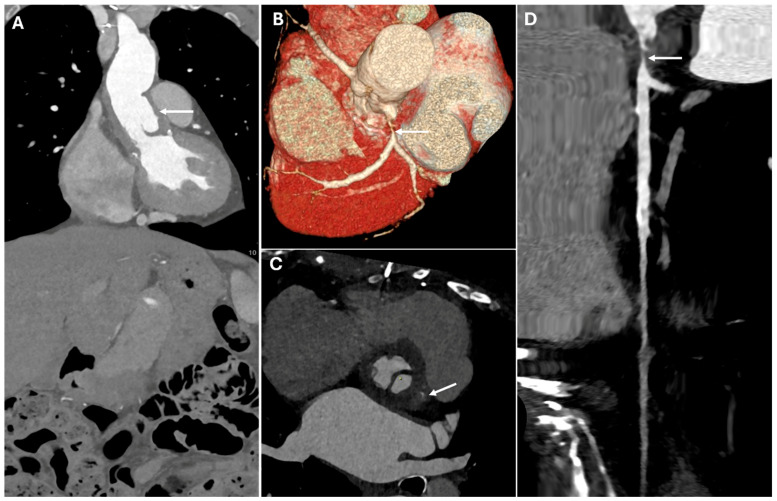
CT images of coronary artery complication. These images illustrate a critical postoperative coronary complication. The image demonstrates malposition of the aortic graft (Panel (**A**), white arrow), which contributes to the left main coronary artery stenosis (Panels (**B**–**D**)—white arrows). The narrowing appears as a focal reduction in vessel caliber with diminished contrast opacification, indicating impaired perfusion.

Coronary artery complications can result from coronary ostial obstruction, kinking, or malpositioning of the coronary button reimplantation during aortic root replacement. Acute coronary ischemia may arise due to technical errors during surgery, leading to myocardial infarction. CTA demonstrates coronary ostial narrowing or complete occlusion with diminished contrast opacification in the affected artery (Figure 5).

**Figure 6:**
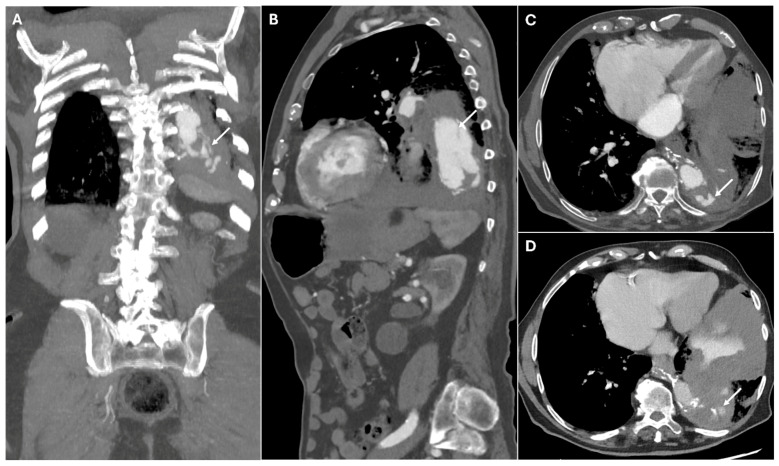
CT images of graft dehiscence. Panels (**A**,**B**) show axial and sagittal CTA images demonstrating dehiscence of the distal anastomosis of the thoracic aortic graft (white arrows). The disrupted graft connection results in direct communication between the vascular lumen and the surrounding mediastinal structures, revealing contrast extravasation into the left lung parenchyma (Panels (**C**,**D**)—white arrows).

Graft dehiscence and leaks occur when there is separation of the graft from the native aorta, often leading to catastrophic hemorrhage. They result from poor anastomotic integrity, infection, or excessive mechanical stress, particularly in patients with underlying connective tissue disorders or vasculitis. They can occur at any anastomotic site or cannulation point. If not promptly diagnosed, they can lead to free rupture, which is often fatal. CTA identifies contrast extravasation at the graft anastomotic site, appearing as active leakage into the mediastinum. A widened mediastinum, fluid collections, and disrupted graft margins are also suggestive of dehiscence (Figure 6).

**Figure 7:**
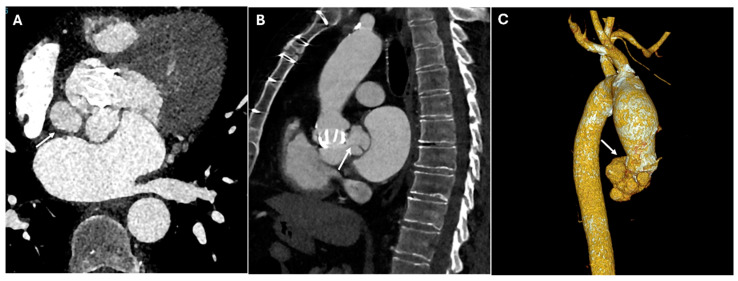
CT images of paravalvular leaks around the prosthetic aortic valve. CT images illustrate paravalvular leaks around the prosthetic aortic valve, in axial (Panel (**A**)), sagittal (Panel (**B**)), and volume rendering (Panel (**C**)) views. The images highlight the areas of contrast extravasation along the prosthetic valve annulus, indicating leakage due to incomplete sealing between the valve and the aortic wall (white arrows).

Paravalvular leaks are identified by contrast extravasation around the prosthetic valve annulus, often appearing as an abnormal jet of contrast outside the expected lumen, which may be best visualized in delayed imaging phases (Figure 7).

**Figure 8:**
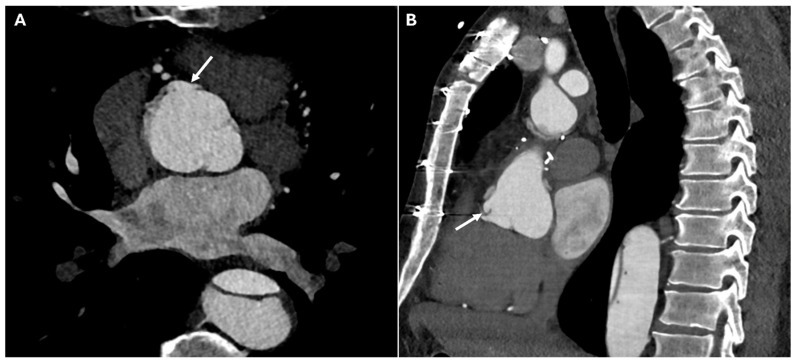
CT images of pseudoaneurysm at the proximal anastomotic site. Panels (**A**,**B**) highlight the presence of a localized, contained pseudoaneurysm in the vessel wall at the site where the proximal graft is sutured to the ascending aorta, characterized by an outpouching of surrounding blood (white arrows).

Pseudoaneurysms develop when there is a contained rupture at an anastomotic site, leading to a contrast-filled outpouching with no true vascular wall. They arise due to suture failure, infection, or chronic mechanical stress at graft interfaces. CTA reveals a contrast-filled sac adjacent to the surgical graft, often with a narrow neck communicating with the vessel lumen. Unlike true aneurysms, pseudoaneurysms lack an intact vascular wall and may contain thrombus or irregular contrast pooling. In postoperative cases, pseudoaneurysms commonly arise at anastomotic sites, points of cannulation, or arteriotomies, including those in the left ventricular outflow tract (Figure 8).

**Figure 9:**
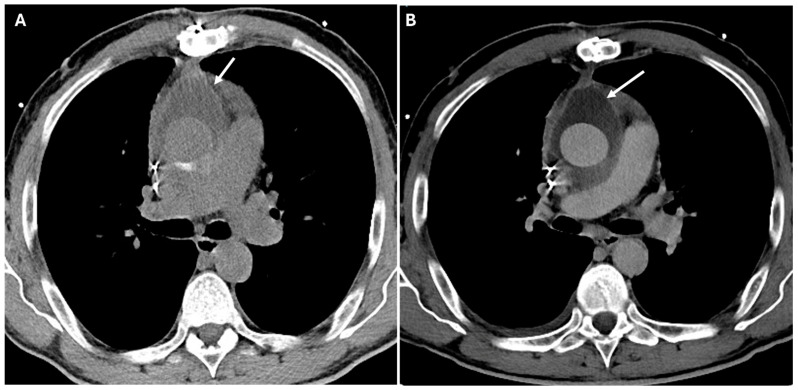
CT images of infection of perigraft fluid collections. Panels (**A**,**B**) show a seroma with slightly thickened and hyperemic walls near the aortic graft (white arrows) after a Bentall procedure.

Certain postoperative imaging features, such as perigraft fluid collections, soft-tissue stranding, and mediastinal air, are frequently observed and do not necessarily indicate pathology. Mediastinal air, for example, is often linked to surgical drain removal and may persist for several weeks. In subacute cases, a contained hematoma may be seen around the graft, with mass effect on adjacent structures. However, some findings should raise concern for infection, particularly fluid collections with rim enhancement or new or increasing perigraft air (Figure 9). Moreover, large or rapidly accumulating effusions can impair cardiac function, leading to hemodynamic instability. CTA shows a fluid collection in the pericardial sac, which may be simple (homogeneous, low attenuation) or complex (heterogeneous, containing high-density thrombus or debris). If tamponade is present, signs such as compression of the right atrium or right ventricle and bowing of the interventricular septum may be observed [4,30].

Complications of TEVAR include endoleaks, stent graft migration, occlusion, infection and fistulas. Endoleaks appear on CTA as persistent contrast enhancement outside the stent graft lumen. Type I endoleaks occur when there is an inadequate seal at either the proximal or distal attachment sites of the stent graft. A failure to seal at the proximal aortic neck or the distal iliac artery leads to persistent blood flow into the aneurysm sac. Type II endoleaks are the most common type and occur when blood flows retrogradely through the lumbar arteries or inferior mesenteric artery into the aneurysm sac. Type III endoleaks occur when there is a defect in the graft itself, either at the junctions where graft components overlap or due to a tear in the graft material. This type of endoleak can be identified through CT imaging, which will show contrast material leaking directly from the graft. Type IV endoleaks are caused by the porosity of the graft material, which allows blood to leak through the graft wall. Type V endoleaks, also known as endotension, are unique in that they occur without a clear source of blood flow into the aneurysm sac. The aneurysm sac continues to enlarge despite there being no detectable leak, and the exact mechanism behind this phenomenon remains poorly understood.

**Figure 10:**
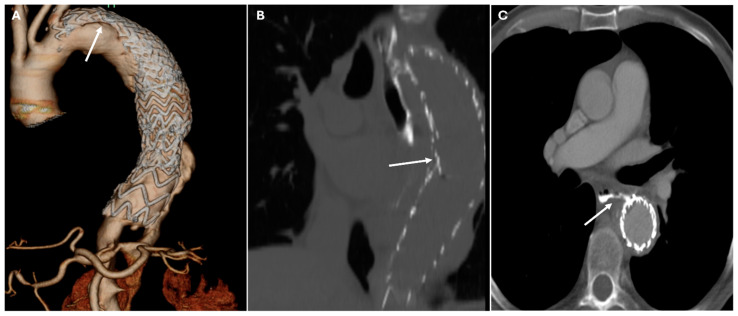
CT images of complications of TEVAR. Panel (**A**) shows the caudal migration of the stent graft in the thoracic descending aorta with an endoleak type IA (white arrow). Panel (**B**) depicts a type III endoleak (white arrow). The endoleak, which occurs at the junction of the stent graft and native aorta, leads to persistent blood flow into the false lumen, causing significant complications such as the aorta-esophageal fistula seen in Panel (**C**) (white arrow). This condition requires urgent intervention due to the risk of hemorrhage and gastrointestinal complications.

Graft migration is defined as displacement of the stent graft > 10 mm from its original position, detected on sequential imaging. Stent graft occlusion appears as a lack of contrast opacification within the graft lumen with potential collateral vessel development. Stent graft infection is suggested by perigraft fluid collections, soft tissue stranding, gas formation, or enhancement of adjacent tissues. Aorto-esophageal or aorto-bronchial fistulas present as direct contrast extravasation into the esophagus or bronchial tree, often with adjacent air or soft tissue thickening (Figure 10). Aorto-bronchial fistulas are abnormal connections between the aorta and the bronchial tree. The most common cause is chronic aortic aneurysms, particularly those that are infected or have undergone aortic dissection. Treatment typically involves a combination of emergency surgery to repair the aorta and control hemorrhage, often through a graft replacement. Aorto-esophageal fistulas, on the other hand, are abnormal connections between the aorta and the esophagus, usually due to the erosion of an aortic aneurysm or from complications following aortic surgery. These fistulas are most commonly associated with aortic arch aneurysms or chronic infections and can lead to massive gastrointestinal bleeding, severe dysphagia, and even septicemia. A combination of aortic repair (either open surgery or endovascular stent grafting) and esophageal repair is typically necessary [30,31].

Table 3 summarizes postoperative complications.

## 8. Surgical Materials in Thoracic Aortic Repair: Imaging Characteristics and Differential Diagnosis of Complications

Surgical grafts used in open repair of the proximal thoracic aorta are primarily made of synthetic polyethylene, while aortic homografts from human donors are rarely used due to their complexity and higher failure rates. These grafts can either replace aortic tissue alone or be shaped into a valvular prosthesis, which is referred to as a composite graft when combined with a valve. On noncontrast CT, synthetic aortic grafts appear to be hyperattenuating relative to the native aorta, though this distinction may be less apparent on postcontrast imaging due to the adjacent blood pool. Structurally, aortic grafts lack the flexibility of native aortic tissue, resulting in a straighter morphology with sharper angulations and, at times, redundant folds. These folds, which are visible on imaging, may create the false impression of dissection when viewed in axial sections. The absence of normal anatomic landmarks, such as the sinotubular junction and the sinuses of Valsalva, is a key feature in identifying grafts, particularly at the aortic root. Subtle caliber changes at the transition between the graft and the native aorta, as well as reinforcement materials, help delineate the extent of the graft.

Endovascular stent grafts, which are frequently employed for aortic repair, consist of a metallic skeleton, often made of nitinol, covered with a polyester graft. The metal framework appears hyperattenuating on a CT scan. Potential mimics include circumferential calcifications. Reinforcement materials, including felt rings, pledgets, sutures, and surgical clips, are frequently used to strengthen vascular anastomoses and prevent complications.

Felt rings, typically composed of tetrafluoroethylene, encircle the aorta at suture sites and appear on CT scans as hyperattenuating linear structures. Their ring-like configuration is best confirmed on orthogonal or volume-rendered images. Felt pledgets, which reinforce vascular access points, appear as small hyperattenuating round or oblong structures along the aorta or arch vessels. Sutures, which also reinforce anastomoses, appear as thin linear hyperattenuating structures on CT and may be associated with small metallic suture anchors, particularly in bioprosthetic aortic valve replacements. Surgical clips, used for vessel occlusion or side-branch graft closure, are highly hyperattenuating, straight-edged, and often produce beam-hardening artifacts. Differentiating these materials from extravasated blood and pseudoaneurysms is essential, and noncontrast imaging plays a crucial role in distinguishing surgical materials, as they are often more conspicuous before contrast administration.

Bioabsorbable hemostatic agents, such as gelatin sponges or oxidized regenerated cellulose, are often used when conventional methods fail to control intraoperative bleeding, particularly in reoperative cases. These materials are generally absorbed within one to two weeks but can persist for longer if used in large quantities. Before absorption, they may appear as heterogeneous masses containing gas, sometimes exhibiting rim enhancement, which can mimic an abscess, hematoma, or retained foreign material. Features that help distinguish these agents from true infection include a linear arrangement of tightly packed gas bubbles that remain stable on serial imaging, as well as the absence of air–fluid levels or a well-defined enhancing wall.

**Figure 11:**
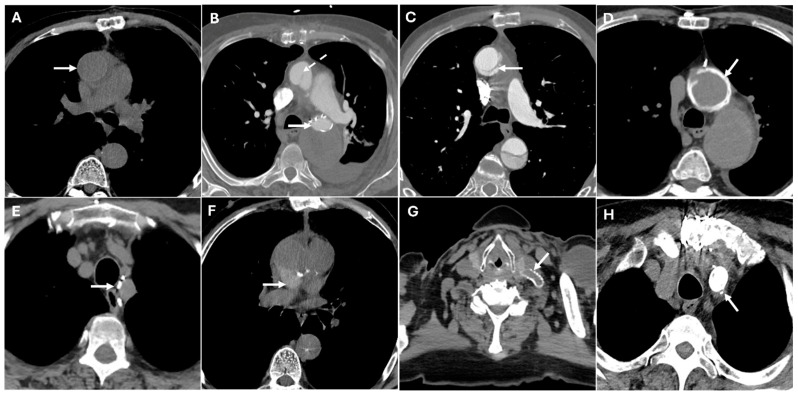
CT images illustrate various surgical materials used in thoracic aortic repair. Panel (**A**) shows the prosthesis of the ascending aorta with slight hyperdensity in the wall (white arrow). Panel (**B**) shows the endograft of the descending aorta, which may mimic aortic calcifications (white arrow), along with an associated fold in the ascending aortic prosthesis that could mimic aortic dissection (dashed white arrow). Panel (**C**) depicts the descending aortic prosthesis with a fold that might simulate an aortic dissection (white arrow). Panel (**D**) shows the surgical suture, which could mimic microcalcifications (white arrow). Panel (**E**) displays surgical suture points, potentially resembling microcalcifications (white arrow). Panel (**F**) shows biodegradable material that could be indicative of a hematoma around the prosthesis (white arrow). Panels (**G**,**H**) illustrate a carotid-subclavian bypass and occlusion of the proximal segment of the subclavian artery, which may mimic calcifications (white arrows).

The left carotid-subclavian graft is often accompanied by occlusion of the proximal left subclavian artery to prevent endoleaks. This occlusion, typically performed with vascular plugs or occluders, appears on CT as hyperattenuating disk- or dumbbell-shaped structures that promote thrombosis. Potential mimics include calcifications [4,29,32]. Figure 11 presents CT images illustrating various surgical materials used in thoracic aortic repair. Table 4 summarizes postoperative surgical materials and the potential differential diagnosis of complications.

## 9. Discussion

Surgical management of thoracic aortic pathology has evolved significantly, integrating open, endovascular, and hybrid techniques to optimize patient outcomes while minimizing surgical risks. Open repair remains the gold standard for complex cases involving the aortic root and ascending aorta, while TEVAR offers a minimally invasive alternative for descending aortic pathology. Hybrid procedures provide tailored solutions for extensive aortic disease, bridging the gap between open and endovascular techniques. Despite these advances, postoperative imaging remains essential, as each approach presents unique imaging characteristics and potential complications.

CTA plays a critical role in both preoperative planning and postoperative surveillance, offering unparalleled anatomical detail for evaluating graft integrity and complications. Differentiating between normal postoperative findings and true pathology is essential to prevent misdiagnosis and ensure timely management of life-threatening conditions. CTA demonstrates a high degree of diagnostic accuracy. The sensitivity of CTA for detecting aortic complications following thoracic aortic surgery is typically 90–95%. The specificity of CT in this context is also strong, ranging from 90 to 98%, indicating that when a complication is detected, CT is highly accurate in confirming it. This level of specificity is important for avoiding false positives, which could lead to unnecessary interventions [25].

Understanding the imaging appearances of surgical materials is crucial for distinguishing expected postoperative changes from complications. Many imaging findings after aortic graft surgery are normal postoperative appearances, but subtle changes can signal complications. For instance, perigraft air is expected within the first weeks; if it increases, persists, or shows rim enhancement, infection should be suspected. Similarly, perigraft fluid is usually thin and homogeneous early on, but if it becomes heterogeneous, septated, or rim-enhancing, further evaluation is warranted. Graft folds and anastomotic bulges are also common and benign when stable and symmetric, but new irregularities or contrast leaks may indicate mechanical failure or pseudoaneurysm. Metallic artifacts are typical near surgical clips, though adjacent tissues must always be evaluated for hidden pathology, such as subtle signs of hemorrhage. Changes in aortic contour or coronary reimplantation sites require special attention: new contour irregularities, mural thrombus, or signs of coronary ischemia are all potential red flags. Table 5 summarizes the red flags to differentiate postoperative imaging findings from complications in thoracic aortic surgery.

**Figure 12:**
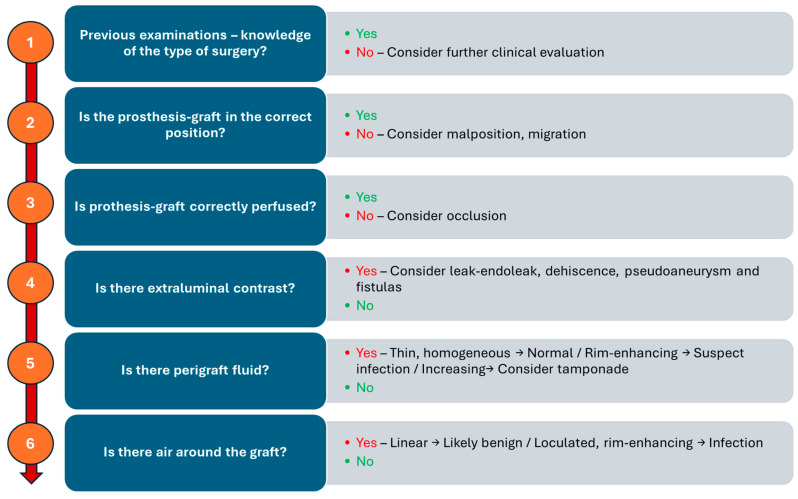
Schematic flowchart for detecting and categorizing potential postoperative complications.

A structured imaging approach allows for early detection of complications, supporting better outcomes and enhancing patient safety. It begins with confirming knowledge of the patient’s surgical history and previous imaging, as this contextual information is crucial for accurate interpretation. The next step is to assess whether the prosthesis graft is in the correct anatomical position; any malposition or migration raises concerns about potential complications. Following this, the graft’s perfusion is evaluated. Finally, the presence of extraluminal contrast, perigraft fluid, and air should be assessed to complete the evaluation. Figure 12 illustrates a possible logical sequence to aid radiologists in systematically detecting and categorizing potential postoperative complications. Moreover, this logic-based framework provides an excellent foundation for structured radiological reporting, which promotes consistency, clarity, and clinical relevance. By following such stepwise protocols, structured reports ensure that critical findings are addressed systematically, reduce variability in interpretation, and facilitate more effective communication with referring clinicians—especially in complex postoperative scenarios.

**Figure 13:**
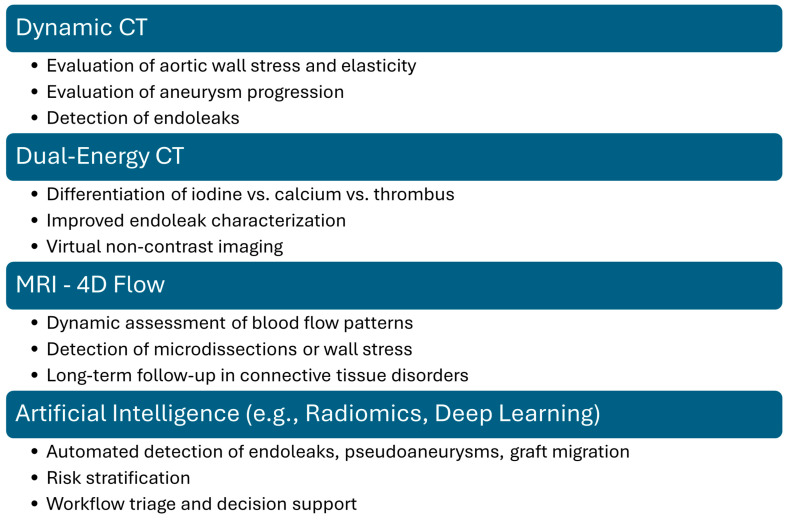
Potential added value of advanced vascular imaging techniques.

Moreover, while CTA remains the gold standard for diagnosing thoracic aortic pathology, advanced imaging techniques such as Dual-Energy CT (DECT), Dynamic CT, magnetic resonance imaging (MRI) and artificial intelligence (IA) offer additional insights into tissue composition, hemodynamic changes, and potential complications. DECT improves vascular imaging by differentiating between iodine contrast, calcium, thrombus, and soft tissue, allowing better visualization of intraluminal thrombi, inflammatory changes, and aortic wall integrity. It also enables virtual noncontrast imaging, reducing radiation exposure in serial follow-ups while enhancing stent visualization by minimizing metallic artifacts. Dynamic CT captures multiple phases of aortic motion, providing valuable information on aortic wall stress, elasticity, and aneurysm progression. It is particularly useful for assessing endoleaks after TEVAR, especially those that may be occult on static CTA [33,34,35]. MRI, particularly magnetic resonance angiography, offers a radiation-free alternative with superior soft tissue contrast, making it ideal for long-term follow-up in younger patients or those with connective tissue disorders. It allows for high-resolution vessel wall imaging to detect inflammatory changes or micro-dissection and provides a comprehensive evaluation of blood flow dynamics through 4D Flow MRI. AI, particularly convolutional neural networks and radiomics, holds potential for improving diagnostic accuracy in postoperative imaging. Preliminary studies show promise in automating the detection of endoleaks and pseudoaneurysms and in distinguishing benign fluid collections from infectious complications. Integration of machine learning into CTA interpretation pipelines may reduce interobserver variability, improve triage in emergency settings, and guide tailored surveillance protocols. Prospective, multi-center studies are needed to validate these tools for clinical use [36,37,38,39]. By integrating these advanced imaging modalities, clinicians can achieve a more comprehensive assessment of thoracic aortic pathology morphology, risk stratification, and post-intervention surveillance. Figure 13 summarizes the potential added values of each advanced imaging technique.

## 10. Conclusions

Thoracic aortic surgery demands meticulous preoperative planning and vigilant postoperative imaging. CTA remains the cornerstone of surveillance, but distinguishing between benign postoperative appearances and life-threatening complications requires nuanced interpretation. This review emphasizes the importance of recognizing imaging mimics and suggests a structured approach for accurate diagnosis.

Integration of advanced imaging techniques offers promising adjuncts to conventional CTA, particularly in complex or ambiguous cases.

Moving forward, collaborative efforts between radiologists, surgeons, and cardiologists are critical not just for treatment, but for refining diagnostic accuracy and optimizing long-term outcomes for patients with thoracic aortic disease, ultimately improving survival rates and quality of life.

## Figures and Tables

**Table 1:** The Ishimaru classification system.

Zone	Anatomical Location	Key Considerations	Preferred Treatment Approach
**0**	Ascending aorta and origin of the brachiocephalic artery	High-pressure region, difficult for endovascular repair	Open surgical repair (e.g., Bentall, David, Cabrol procedures)
**1**	Aortic arch with the origin of the left common carotid artery	Stent placement may require extraanatomical revascularization	Extraanatomical revascularization using the right common carotid artery or right subclavian artery as the donor vessels.
**2**	Aortic arch with the origin of the left subclavian arteries	Critical location for arch vessel perfusion	Extraanatomical revascularization involves left carotid-subclavian bypass or transposition.
**3**	Proximal descending thoracic aorta (distal to the left subclavian artery)	Common landing zone for TEVAR	TEVAR
**4**	Distal descending thoracic aorta, extending toward the diaphragm	Can be treated with stent grafts or open repair for complex disease	TEVAR or open surgical repair depending on complexity

Abbreviations—TEVAR: thoracic endovascular aortic repair.

**Table 2:** Surgical management of the thoracic aorta and CT findings.

Procedure	Indication	Surgical Approach	CT Imaging Characteristics
Bentall Procedure	Aortic root aneurysms, Type A dissection	Composite graft with valve, coronary reimplantation	Tubular graft replacing aortic root, prosthetic valve
Cabrol Procedure	Complex reoperations, severe atherosclerosis	Prosthetic conduit for coronary artery reimplantation	Graft with parallel interposed conduit
Yacoub Procedure	Aortic root aneurysms, valve-sparing repair	Synthetic graft remodeling sinuses of Valsalva	Scalloped aortic root graft with preserved valve
David Procedure	Marfan syndrome, younger patients	Valve-sparing root replacement with graft	Native valve reimplanted into supportive graft
Hemiarch Repair	Limited aortic arch pathology	Zone 0 reconstruction with lesser curvature graft	Graft in the aortic hemiarch
Hybrid Type I Repair	Extensive arch disease	Arch debranching, stent landing in zone 0	Endovascular stent plus revascularized arch vessels
Hybrid Type II Repair	Extensive arch disease	Arch debranching, open surgery and stent landing in zone 0	Tubular surgical graft with high-density metallic stent
Hybrid Type III Repair	Extensive arch disease	Open graft extending to descending aorta, endovascular stent	Tubular surgical graft with high-density metallic stent
Elephant Trunk Technique	Staged repair of extensive aortic disease	Open arch graft extending into descending aorta	Floating tubular graft within descending aorta
Frozen Elephant Trunk	Single-stage aortic arch + descending repair	Endovascular stent incorporated within open graft	Seamless transition between proximal graft and distal stent
Reverse Elephant Trunk	Staged descending arch repair	TEVAR followed by open arch surgery	Combination of open and endovascular grafts
Buffalo Trunk Technique	Combined arch and descending aorta repair	Open arch graft with endovascular stent	Hybrid repair with visible proximal graft and extended stent
TEVAR	Descending aortic aneurysms, dissections	Endovascular stent placement	High-density metallic structure conforming to aortic lumen

Abbreviations—TEVAR: thoracic endovascular aortic repair.

**Table 3:** Postoperative complications in thoracic aortic surgery and CT findings.

Complication	Cause	CT Imaging Features	Clinical Relevance
**Coronary Artery Complications**	Coronary ostial obstruction	Narrowed or occluded ostium, diminished contrast opacification	Can lead to myocardial infarction
**Graft Dehiscence and Leaks**	Poor anastomotic integrity, infection, mechanical stress	Contrast extravasation at anastomotic sites, widened mediastinum, disrupted graft margins	Massive hemorrhage
**Paravalvular Leak**	Suture failure, prosthesis malalignment	Contrast extravasation around prosthetic valve annulus	High rupture risk
**Pseudoaneurysm**	Suture failure, infection, chronic mechanical stress	Contrast-filled sac adjacent to graft, narrow neck	High rupture risk
**Perigraft Fluid Collection and Infection**	Post-surgical infection	Rim-enhancing fluid collections, intrinsic air	Can progress to graft infection and sepsis
**Pericardial Effusion and Tamponade**	Post-surgical tamponade	Fluid collection in pericardial sac, cardiac chamber compression, septal bowing	May impair cardiac function
**Endoleaks (TEVAR)**	Incomplete sealing, graft migration	Persistent contrast enhancement outside stent graft lumen	Can lead to aneurysm expansion and rupture
**Graft Migration (TEVAR)**	Insufficient landing zone, poor fixation	Displacement > 10 mm on sequential imaging	May cause endoleaks or malperfusion
**Graft Occlusion (TEVAR)**	Thrombosis, intimal hyperplasia	Lack of contrast opacification within the graft, collateral vessel formation	May result in ischemia
**Graft Infection (TEVAR)**	Post-surgical infection	Perigraft fluid collections, soft tissue stranding, gas formation, or enhancement of adjacent tissues	Can progress to graft infection and sepsis
**Aorto-Esophageal or Aorto-Bronchial Fistulas**	Erosion of graft into adjacent structures	Contrast extravasation into esophagus or bronchial tree, adjacent air	Massive hemorrhage

Abbreviations—TEVAR: thoracic endovascular aortic repair.

**Table 4:** Postoperative surgical materials and the potential differential diagnosis of complications.

Category	Characteristics	CT Details	Potential Differential Diagnosis
**Synthetic Grafts**	Made of synthetic polyethylene.	Hyperattenuating relative to native aorta on noncontrast CT. Less apparent on postcontrast imaging due to adjacent blood pool. Lack flexibility, leading to straighter morphology and sharper angulations.	Sharper angulations and redundant folds may mimic dissection.
**Stent grafts**	Consist of a metallic skeleton covered with polyester. May include circumferential metallic rings or fenestrations for arch vessels.	Metallic framework appears hyperattenuating on CT.	Mimic calcifications or foreign bodies.
**Reinforcement Materials**	Felt rings, pledgets, sutures, and surgical clips used to strengthen anastomoses.	Hyperattenuating with beam-hardening artifacts.	Mimic calcifications, foreign bodies or pseudoaneurysms.
**Bioabsorbable Hemostatic Agents**	Used for intraoperative bleeding control.	Heterogeneous masses No air–fluid levels or enhancing walls. Linear arrangement of gas bubbles helps distinguish from infection.	Mimic abscesses, hematomas, tumors or retained foreign bodies.
**Carotid-Carotid Bypass Grafts**	Left carotid-subclavian graft often accompanied by proximal left subclavian artery occlusion.	Occlusion appears as hyperattenuating disk- or dumbbell-shaped structures. Grafts visible on multiplanar reconstructions.	Mimic calcifications

**Table 5:** Red flags for suspicious complications after thoracic aortic surgery.

Imaging Feature	Expected Postoperative Appearance	Red Flags Suspicious for Complication
Perigraft air (early)	Present < 2–3 weeks post-op; stable	Increasing, rim-enhancing, or delayed presence
Perigraft fluid	Thin, homogeneous, no enhancement	Heterogeneous, rim-enhancing, septated
Graft folds	Linear, symmetric, stable on serial CTA	New, irregular, or associated with contrast leak
Anastomotic bulge	Small, smooth, uniform	Saccular, eccentric, narrow-necked (suggests pseudoaneurysm)
Metallic artifact	Beam hardening near clips/sutures	No diagnostic value alone—evaluate adjacent structures
Aortic contour	Straightened, with minor caliber changes at graft interface	Irregular contour, mural thrombus, or new hematoma
Coronary reimplantation site	Well-positioned, opacified coronaries	Focal stenosis, absent enhancement, infarct signs

## Data Availability

The data are contained within the article.
